# Supplementary material for: Invasive potential of tropical fruit flies in temperate regions under climate change
Source: Commun Biol. 2021 Sep 30;4:1141. doi: 10.1038/s42003-021-02599-9 (PMC8484444; doi:10.1038/s42003-021-02599-9)
Supplement: Supplementary file 2 — Supplementary Information [file 42003_2021_2599_MOESM2_ESM.docx]

**Supplemental Information**

**Invasive potential of tropical fruit flies in temperate regions under climate change**

Andrew Paul Gutierrez, Luigi Ponti, Markus Neteler, David Maxwell Suckling,

José Ricardo Cure

ORCID registry:

Andrew Paul Gutierrez: <https://orcid.org/0000-0001-7773-1715>

Luigi Ponti: <https://orcid.org/0000-0003-4972-8265>

Markus Neteler <https://orcid.org/0000-0003-1916-1966>

David Maxwell Suckling <https://orcid.org/0000-0001-7216-9348>

José Ricardo Cure <https://orcid.org/0000-0001-5816-0259>

**Table of Contents**

**Mediterranean fruit fly**

Biology and prospective distribution

**Melon fruit fly**

Biology and prospective distribution

**Oriental fruit fly**

Biology, analysis of CDFA detection data, and prospective distribution

**Mexican fruit fly**

Biology and prospective distribution

**Climate change effects on the geographic distribution and relative abundance of the four tropical fruit flies**

**Discussion of the distributed maturation time model**

**Summary of data used to parameterize the BDFs:**

Data extracted from the literature used to parameterize the BDFs for each of the species are illustrated in text Figures and summarised in attached Excel files. Figures without data symbols in the text were estimated from text or figures the cited the literature. Note that temperatures are not listed in sequence, indicating different sources for the data subsets. Data values shaded red are suspect values and were not used in fitting BDFs.

**Mediterranean fruit fly**

The highly polyphagous medfly is of sub-Saharan Africa^1^ and is a serious pest in many topical regions of the world and in some Mediterranean climate areas. It was first reported in Costa Rica in 1955, moved progressively northward, and infested virtually all Central American countries^2^. As part of an ongoing sterile insect technology (SIT) eradication program, tripart agreements between Guatemala, Mexico, and the USA (years 1975, 1981, 2015) led to the development of a mass rearing facility for the production of sterilized medfly (and other fruit flies) at Metapa, Chiapas in southern Mexico. The first sterile medfly releases began in 1978, with the facility currently producing in excess of 1.3 billion sterile flies per week to service the ongoing eradication program in Guatemala and Mexico, to prevent its invasion northward to the USA, and to eradicate incipient infestations in Mexico and the USA^3^. Medfly infestations in the USA have been recorded primarily in Florida, and in the Los Angeles Basin and south San Francisco Bay area of California in the late 1970s and 1980s (e.g., refs.^4^). In sharp contrast to the host specific olive fly, the polyphagous medfly has not established in California (Dr. Kyle Beucke, Primary State Entomologist, California Department of Food and Agriculture (CDFA), personal communication), though some claim establishment at undetectable levels^5^ (see ref. ^6^). Chemical and SIT eradication programs are posited responsible for eradicating incipient infestations^6,7^.

**Prospective distribution of medfly -** The PBDM predicts moderate favorability for medfly in Central America, and absent eradication efforts, large areas of Mexico. The extensive arid desert areas of northern Mexico provide a natural barrier to invasion of the USA (Fig. S1a). With a climatic favorability index of <0.5, incipient medfly populations could develop during favorable periods of the year, but not persist at detectable levels in the Los Angeles Basin (inset figure in Fig. S1a)^5^. The rest of California and the desert Southwest are unfavorable (see ^4^). This contrasts with the Carey et al.^8^ supposition that large areas of California are “medfly friendly” based on the generalization that they are similar to climes at the same latitudes in the Mediterranean Basin where medfly populations occur (e.g., Italy, coastal Croatia and Southern Greece, and coastal Israel/Palestine; Fig. S1c). What this fails to recognize is that weather in temperate areas of California may be limiting during different times of the year due to cold and/or hot temperatures (e.g., the great Central Valley). Szyniszewska et al.^9^ used CLIMEX and MED-FOES models to examine the most favorable periods for medfly infestations at specific locations in California and Florida, but the analysis did not answer whether establishment could occur. We used the number of predicted pupae per year in North and Central America as a measure of favorability, finding an inverse correlation to the coefficient of variation (Fig. S1b) as a percent (i.e., $medfly\text{ }pupae=7.298.9e^{-0.037CV},R^{2}=0.87$). [N.B. Similar trends are also found for the other three species in our study.]


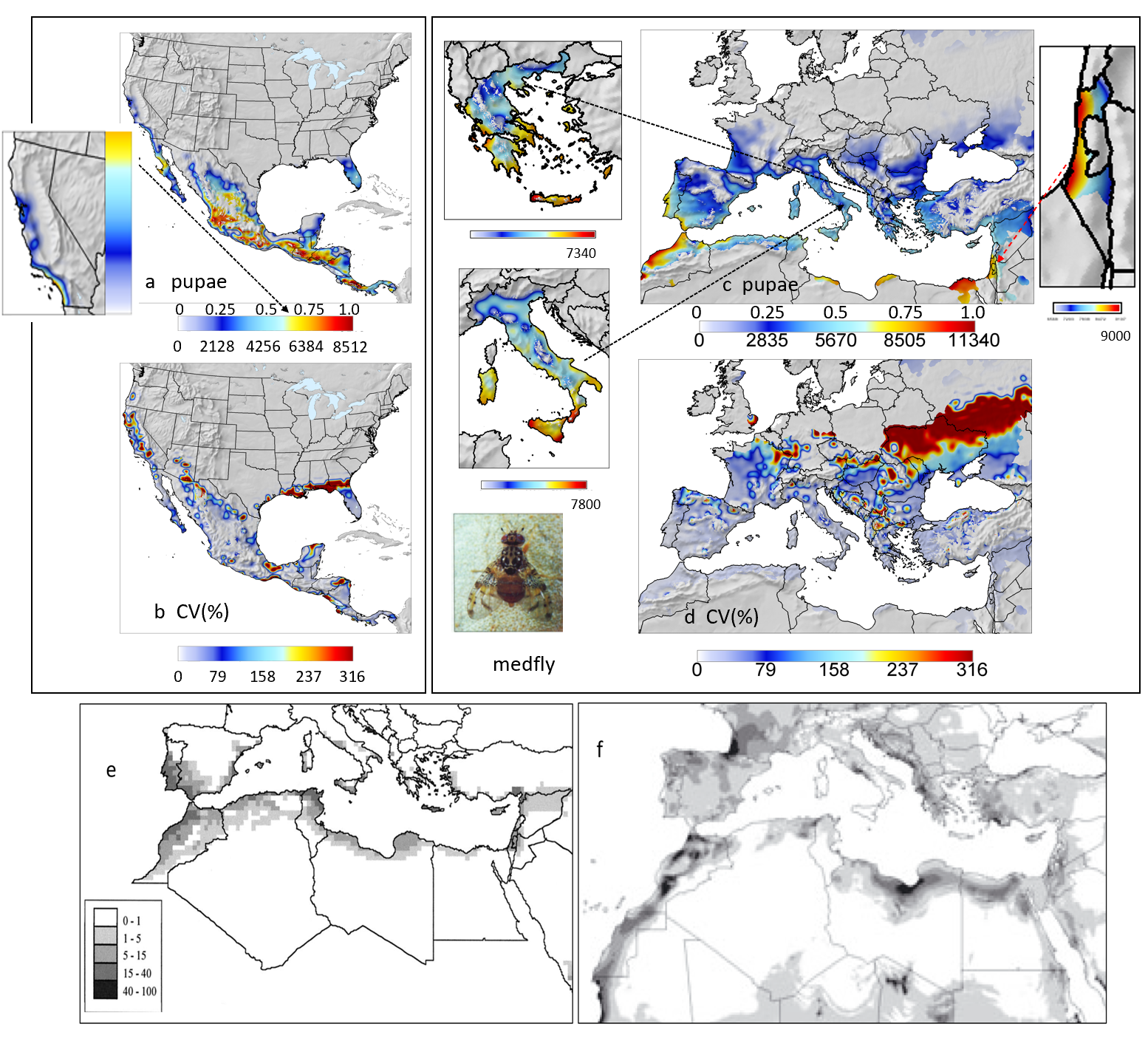


Figure S1. Average predicted distribution of **Mediterranean fruit fly** in North and Central America (NA-CA) and the European-Mediterranean region (E-M) for the period 1980-1990: (NA-CA) (a) sum of annual pupae and (b) CV(%), and (E-M) (c) sum of annual pupae with inset figures for Greece, Italy and Israel, and (d) CV(%). Insets are (e) from Vera et al.^10^ (CLIMEX) and (f) from De Meyer et al.^1^ (Principal Components Analysis). Clip of Mediterranean fruit fly was taken from a photograph by Jack Kelly Clark provided courtesy of the University of California Statewide IPM Program.

Medfly has established in limited areas of the European-Mediterranean region. Fig. S1c shows the prospective distribution of high favorable areas for medfly population development and establishment (coastal and near coastal areas Morocco, the Nile Delta, and the Levant (Fig. S1c). Inset maps with separate density scales suggest favorability for areas of Italy, Greece, and Israel/Palestine, while more inland areas (e.g., of Croatia, Serbia, Albania, Northern Greece, Turkey) are marginal to unfavorable due to cold weather. Israely et al.^11^ found that medfly does not readily overwinter in the Judean Hill and re-infestation occurs annually. Immature stages have been found to survive the winter period in unharvested fruit in Israel^12^, northern Greece^13^, and in fruit cellars in Lombardy, Italy ^14^.

Vera et al.^10^ used the climate envelope model CLIMEX to infer the climatic requirements of medfly from its observed geographical distribution in the Mediterranean region. Our prospective distribution of medfly based solely on the biology agrees with their coarser grain correlative CLIMEX projections for much of the Mediterranean Basin, but not in the hotter drier inland areas of North Africa and the Middle East (Fig. S1e). The relatively good comparisons are likely due to the fact that the growth indices of CLIMEX have their origins in the physiological indices of Fitzpatrick and Nix^15^ and Gutierrez et al.^16^ that were used initially to develop CLIMEX, and that are components of our model (i.e., the concave scalars for reproduction due to temperature and relative humidity (see text Fig. 1d,e)). The predictions of the geographic distribution of De Meyer et al.^1^ based on Principal Components Analysis of occurrence data are much more expansive and project into areas not covered by our analysis (Fig. S1f).

**Melon fly**

Melon fly is originally from India, but it is now widespread through much of the Southern Asia, neighboring islands, and in sub-Saharan Africa (<https://www.aphis.usda.gov/>). It was the first tephritid fruit fly species to establish in Hawaii, after its accidental introduction from Japan around 1895 (<http://www.extento.hawaii.edu/kbase/crop/Type/bactro_c.htm>). Infestations were found in the mainland USA in Florida in 2002 and 2015, with more than 30 detections in California (CDFA data^5^).

**Prospective distribution of melon fly -** The most favorable areas are the humid tropical areas of Mexico and Central America, with moderate favorability in south Florida. Important factors are the right biased scalars for temperature and relative humidity on adult reproduction (text Figs. 1 and 6). The PBDM predicts lower favorability in NE Mexico, south Texas, across the southern part of the gulf states (FI<0.5), and northern Florida (0. 5 < Index <0.75), while conditions in California’s cooler coast and the hot-dry agriculturally rich Great Central Valley are predicted unfavorable (Figs. S2a-d). The isoline FI=0.5 is shown in white (Fig. S2a), below the average cumulative annual cold weather mortality $\bar{\mu}_{<7.95^{\circ}C}$= 1 (data clipped < 3; Fig. S2b). Favorability to average hot weather is not clearly defined except in desert areas of NW Mexico, SW Arizona, and SE California (Fig. S2c). We note that large areas of eastern Mexico and south Texas are characterized by high variability of hot weather during the year resulting in high $CV_{\bar{\mu}_{>34^{\circ}C}}$>70% (Fig. S2d).


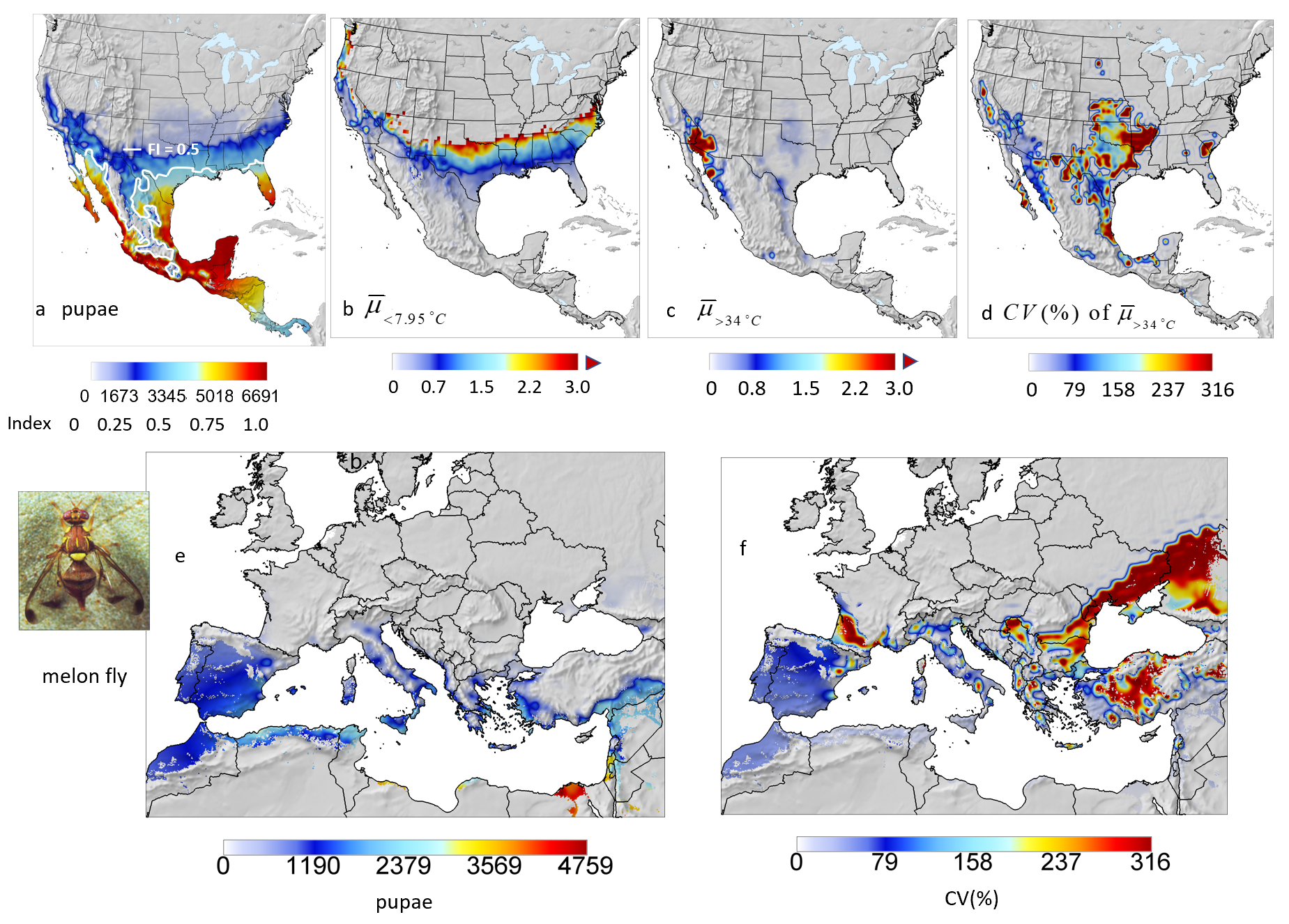


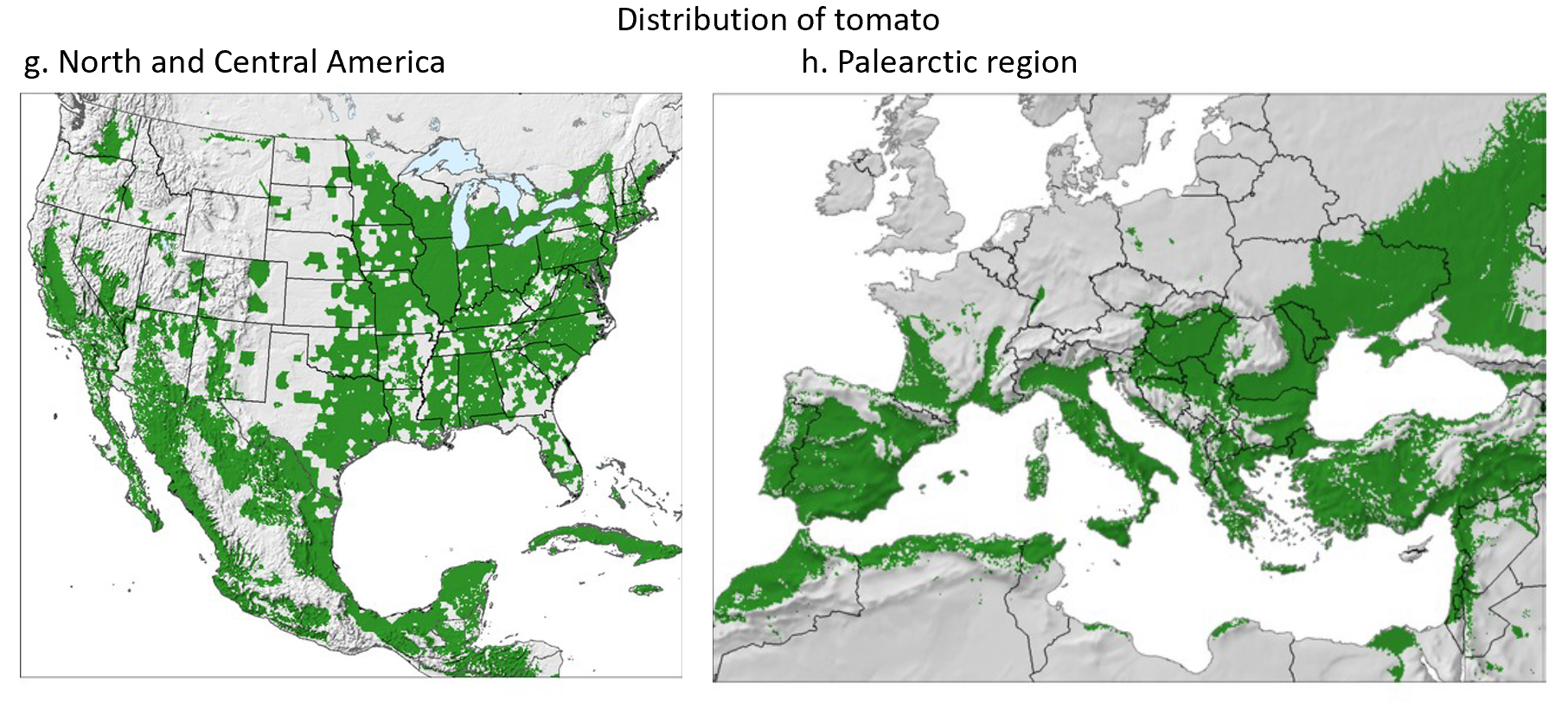


Figure S2. Average prospective distribution of **melon fruit fly** in North and Central America (NA-CA) and the European-Mediterranean region (E-M) for the period 1980-1990: (NA-CA) (a) sum of annual pupae (NA-CA), (b) average annual $\bar{\mu}_{<7.95^{\circ}C}$ (NA-CA), (c) average annual $\bar{\mu}_{>34^{\circ}C}$ and (d) its annual CV(%), and E-M (e) sum of annual pupae, (f) CV(%) of pupal densities, and the distribution of tropical and temperate tomato in (g) North-Central America and in the (h) the Palearctic region respectively (Climate-limited tomato distribution are from the Global Agro‐ecological Zones (GAEZ v3.0) dataset (IIASA and FAO 2012) (<http://www.fao.org/nr/gaez/en/)>). IIASA II for ASA, FAO F and AO of the UN (2012) Global Agro-ecological Zones (GAEZ v3.0). IIASA, Laxenburg, Austria and FAO Rome, Italy. Clip of melon fly was taken from a photograph by Jack Kelly Clark provided courtesy of the University of California Statewide IPM Program.

Melon fly has a sparse prospective distribution in the European-Mediterranean region (Fig. S2e), with low coefficient of variability in favorable areas (Fig. S2f). The maximum number of pupae in favorable areas is about half the densities predicted for Mexico and Central America.

The fly attacks a wide variety of hosts, and is commonly associated with melon, cucumber, and tomato. Figs. S2g, h shows the extensive distribution of tomato in North-Central America and the Palearctic region.

**Oriental fruit fly**

The oriental fruit fly is native to tropical Asia where it is a destructive pest of fruit and vegetables^17^. It is established over much of tropical sub-Saharan Africa^18^, and on all major Hawaiian Islands after being accidentally introduced in the mid-1940s, and likely earlier^19^. It has been detected in Florida in 2002 and 2015, and annually in California during 1960-2012 at various locations, especially in the Los Angeles Basin (total adult flies 2,636 captured), lower numbers in the San Diego area (1,262) with an apparent outbreak in 1974 (i.e., 979 flies captured), and in the San Francisco Bay area (327 flies) (Fig. S3) (see CDFA data reported by Papadopoulos et al. and Zhao et al. ^5, 20^). None of the paired linear regressions of total annual oriental flies detected at the three locations during 1960-2020 (data from Fig. S3) were significant (R^2^ < 0.01). The infestations are posited to have been eradicated (https:// www.aphis.usda.gov/ aphis/ resources/ pests-diseases/). Because the oriental fruit flies were detected so often in California, we examine some of the abiotic factors that may limit its establishment.


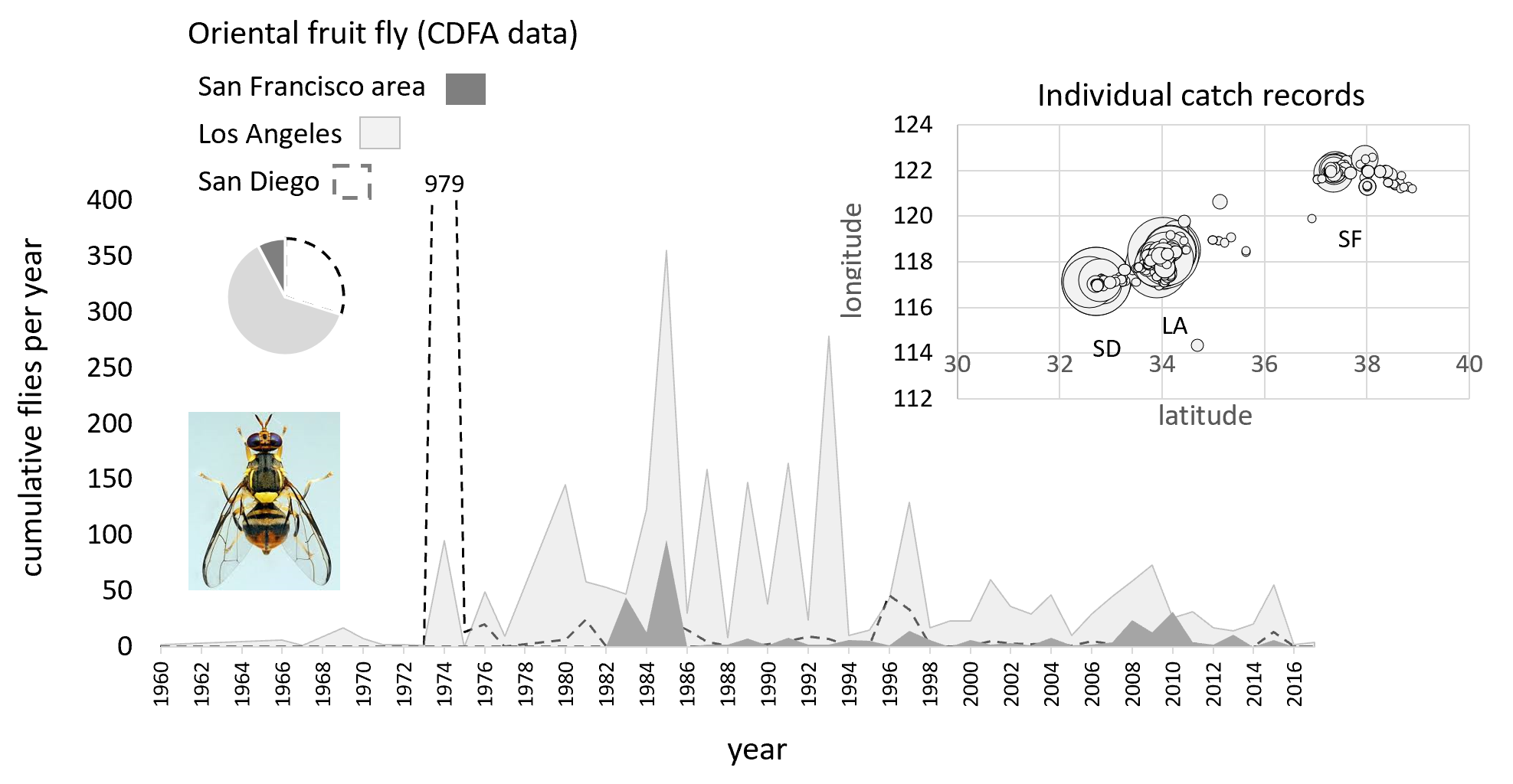


Figure S3. Summary of California Department of Agriculture (CDFA) **oriental fruit fly** detection data as number of flies plotted on years, with a relative comparison of catches (size of circles) in the upper right corner insert. Individual occurrences (number of circles), with the smallest circles being of unit 1 and the largest being 79. The number of traps during each year is not known. Clip of the oriental fruit fly was taken from a photograph by Jack Kelly Clark provided courtesy of the University of California Statewide IPM Program.

Linear regressions of the numbers of adults trapped per year on cumulative annual cold weather mortality ($\bar{\mu}_{\text{<8.87}^{\circ}\text{C}}$, see Fig. 4a), or on cumulative degree days <8.87°C (see Fig. 4b) were not significant (R^2^ < 0.05). The trends for cumulative annual degree days are increasing at all three locations indicating that temperatures are increasing (S4b, climate change), with strong correlations between San Diego and San Jose to the Los Angeles data (S4c). Los Angeles has on average 12% fewer degree days than San Diego, but 27% more than San Jose. Furthermore, plots of daily mean temperatures on daily %RH for Los Angeles, with the oviposition scalars (*φ(T), φ(RH)*) super imposed suggest that the optimal conditions for the fly (i.e., symbol • at *φ(T)^opt^* = 1 and *φ(RH) ^opt^* =1) occur near the margin of observed temperatures (Fig. S4d). This figure suggests that oriental fruit fly invading during warmer periods could breed, and F1 and later adult flies could be detected. However, because reproduction is affected by both temperature and RH, the oriental fruit fly would not survive some relatively mild winter in Los Angeles, and certainly not colder yet mild winters in San Jose, CA just south of San Francisco (Fig. 4a). Simulations for Los Angeles during 1980-2020 (Fig. S4e) illustrate the effect of scalars *φ(T)* singly and in combination with the compounding important effects of low *φ(RH)*) on cumulative annual pupae. The result further suggests that the time interval chosen for specific and region wide studies can affect the relative magnitude and the distribution of mapped favorability (e.g., Fig. S4e).


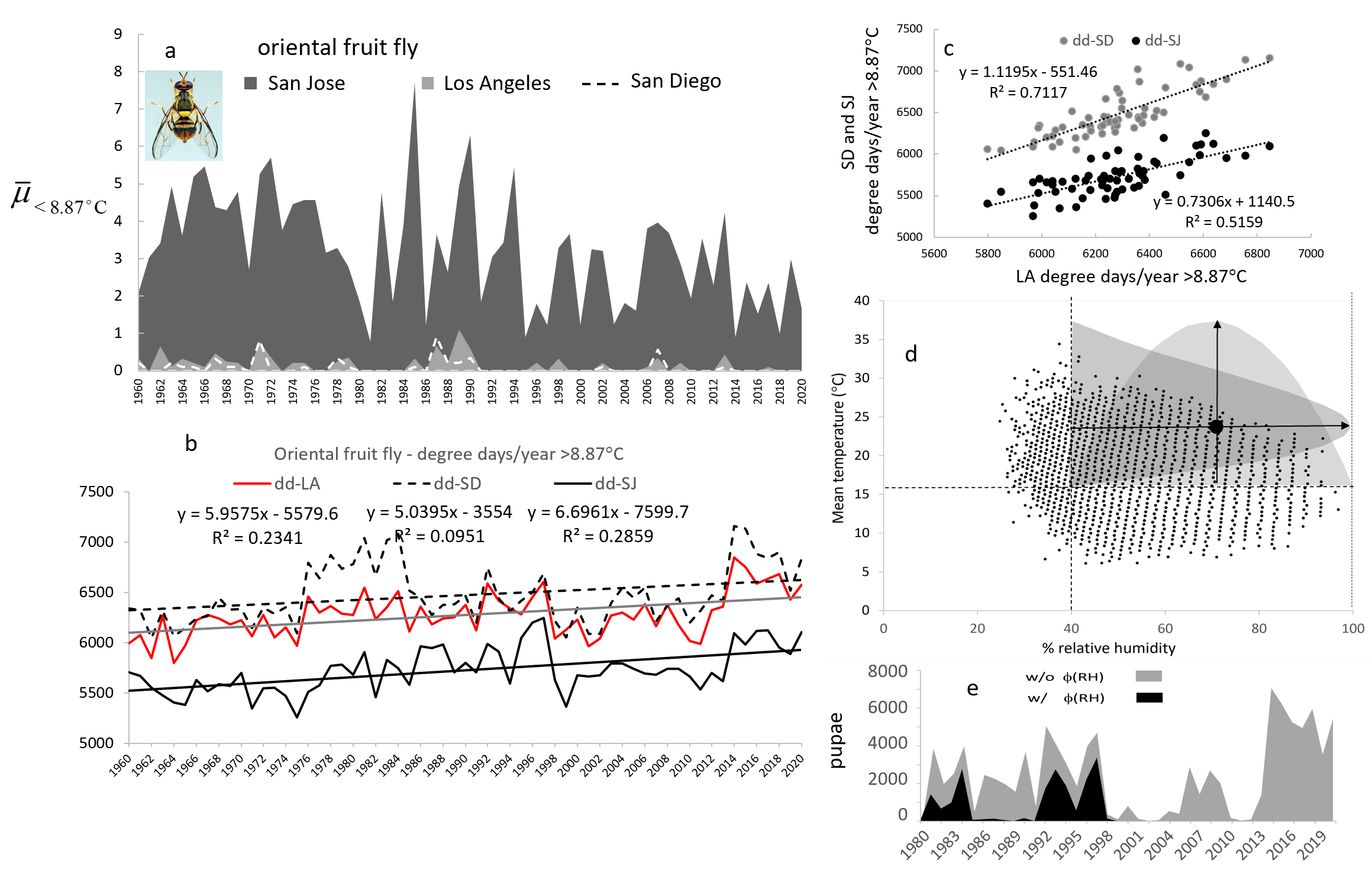


Figure S4. Abiotic factors affecting **oriental fruit fly** at three locations in coastal southern California:

(a) illustrates the cumulative annual cold weather mortality rates for oriental fruit fly ($\bar{\mu}_{\text{<8.87}^{\circ}\text{C}}$, see text) for Los Angeles, San Diego and San Jose, CA, (b) shows the cumulative annual degree days <8.87°C and the apparent effects of climate warming (i.e., the positive slope), (c) shows the correlations of cumulative degree days at San Jose and San Diego on cumulative degree days in Los Angeles, (d) plots of Los Angeles daily mean temperatures on daily % relative humidity with normalized BDFs scalars for temperature (*φ(T)*) and % RH *(φ(RH)*) that scale reproduction superimposed with the optimum *(*(*φ(T)*=1 at *T_opt_, φ(RH)*=1 at *RH_opt_)* indicated by black symbol •), and (e) the simulated annual number of pupae for Los Angeles, CA for the period 1980-2020 with the effect of *φ(T)* and *φ(RH)* ( black) and without the effect of *φ(RH)* (grey). Clip of oriental fruit fly was taken from a photograph by Jack Kelly Clark provided courtesy of the University of California Statewide IPM Program.

**Prospective distribution of oriental fruit fly -** The prospective distribution of oriental fruit fly in North America is primarily south-central Mexico, with decreasing favorability northward around the near coastal regions of Texas and the gulf states (FI < 0.5), with higher favorability in central to south Florida (FI ~0.75) (Fig. S5a). Note that areas with predicted high fly densities have low CV (Fig. S5b). Low relatively humidity and high temperature during summer restrict the fly’s reproduction that is skewed to the lower part of its favorable temperature range (text Figs. 1 and 7). High temperatures and low RH during summer would limit reproduction in the Great Central valley of California, the desert regions of Arizona and northern Mexico, and lower reproduction in the southern reaches of Central America where temperatures are consistently above the oviposition optimum of ~24°C (see text Fig. 1, Fig. S4d). Cumulative average annual cold weather daily mortality rates were truncated at $\bar{\mu}_{\text{<8.87}^{\circ}\text{C}}$= 5 (see text and Fig. S5c), and suggests the northern limits of favorability is mean annual sum $\bar{\mu}_{\text{<8.87}^{\circ}\text{C}}$~ 1.2, with low RH limiting the fly in arid regions of Mexico and the USA (e.g., Fig. S4e). Removing lattice cells data with cumulative pupae < 10 (i.e., highly unsuitable areas), a linear multiple regression model (LMR) shows that only mortality due to cold was not significant, while $\bar{\mu}_{>34^{\circ}C}$, mm annual rainfall as a surrogate for RH, and degree days above 10°C as a measure of the length of the favorable season were positive highly significant (*p* < 0.01) (eqn. S1).

[S1]


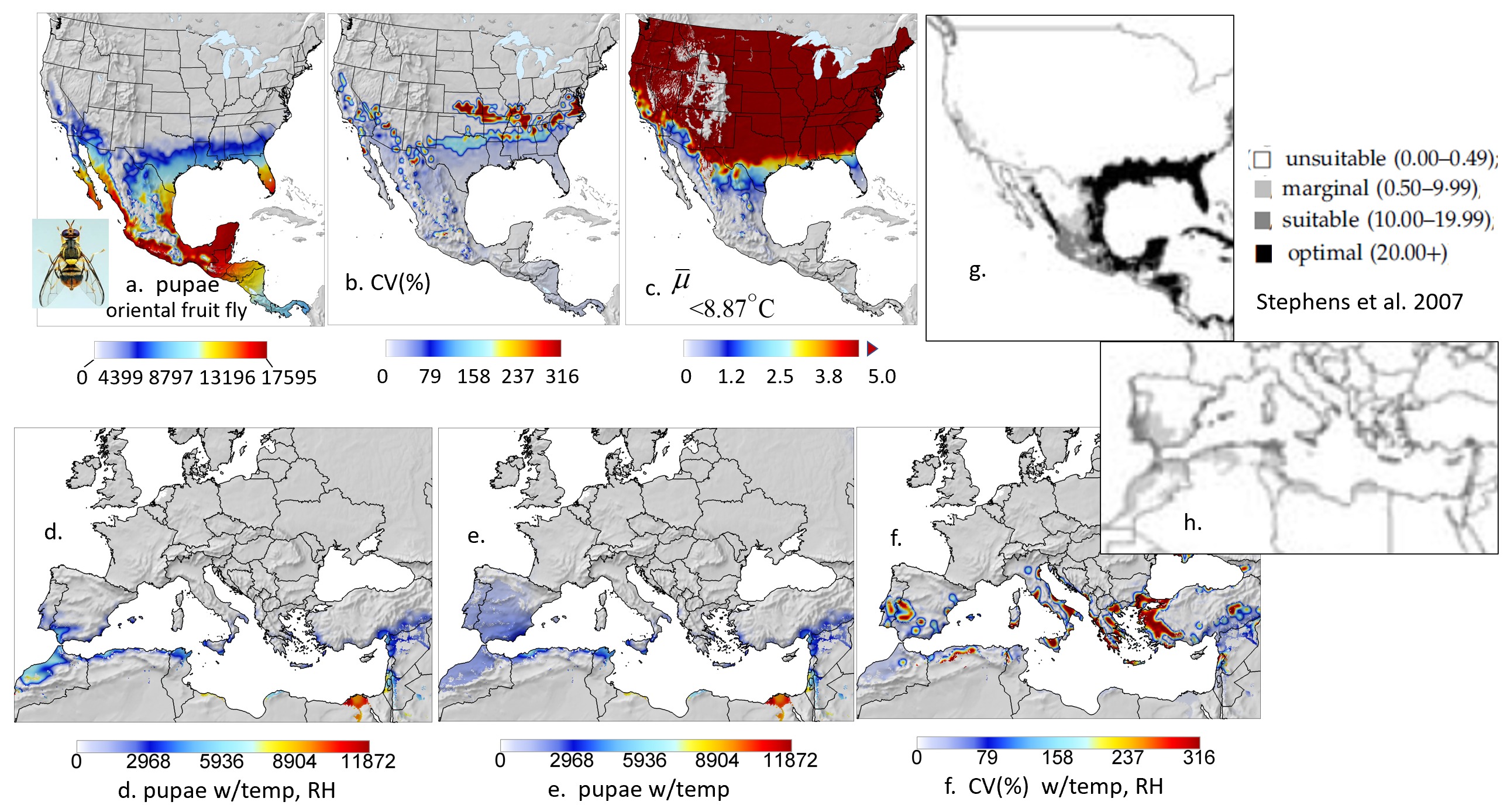


Figure S5. Average prospective distribution of **oriental fruit fly** in North and Central America (NA-CA) and the European-Mediterranean region (E-M) for the period 1980-1990: (NA-CA) (a) sum of annual pupae, (b) CV(%), (c) $\bar{\mu}_{\text{<8.87}^{\circ}\text{C}}$ truncated at 5, and for (E-M), (d) the sum of annual pupae, (e) sum of annual pupae removing the effects of RH, and (f) the CV(%) of pupal data mapped in (d). The insets g and h are the CLIMEX projections of Stephens et al.^21^. Clip of oriental fruit fly was taken from a photograph by Jack Kelly Clark provided courtesy of the University of California Statewide IPM Program.

Our model predicts a distribution similar to that reported by Stephens et al.^21^ using CLIMEX, with lower favorability for North and Central America predicted by our model (Fig. S5a vs. S5g). The differences are most striking for the south eastern USA.

The prospective distribution of the oriental fruit fly in the European-Mediterranean region is greatly restricted (Fig. S5d) with highest favorability being the Nile Delta, with lower favorability along coastal area of North Africa (Morocco, Libya), and south coastal areas of Israel/Palestine with further decreasing favorability northward to southern Turkey and beyond. Small areas of Spain and Sicily are marginally favorable. Most areas of the Palearctic have FI << 0.5. The fly’s distribution in the Mediterranean Basin is limited by both low and high temperatures, and low RH. However, removing the effect of RH changes the geographic range (i.e., the Iberian Peninsula) does not increase the area of suitability or the maximum density (Fig. S5d vs. S5e). The PBDM predicts a very limited distribution of favorability in the European-Mediterranean region similar to that reported by Stephens et al.^21^ (Fig. S5d,e vs. inset S5h) that missed the high favorability of the Nile Delta. A LMR model of the simulation data for the European-Mediterranean region is eqn. S2.

[S2]

All four independent variables are highly significant (*p*<0.01) with the signs of the coefficients being in the expected direction. The effect of cold mortality ($\bar{\mu}_{<8.87^{o}C}$) is 13.4-fold greater than for high temperature mortality ($\bar{\mu}_{>34^{o}C}$).

**Mexican fruit fly**

*Anastrepha* flies are endemic to the new world^22^, with the Mexican fruit fly (*A. ludens*; mexfly) posited to be native to Central America^23^. Mexfly is found from the tropical forests of Costa Rica northward to the semi-arid lands of southern Texas^24^. In Mexico, the fly attacks at least 60 varieties of fruit (e.g., mango), has a close association with Rutaceae such as native sylvatic yellow and white sapote, and with citrus^25,26^. In endemic areas, adult flies are found throughout the year in citrus orchards, even during the dry season when hosts are unavailable^25,27,28^. While no sylvatic reservoir for the fly occurs in the USA, citrus in private and agricultural plantings occur in many areas of Arizona, California, Florida and Texas, and would ensure a year around supply of hosts^25,29^. In tropical areas, rainfall does not significantly impact emergence and survival of adults, with the yearly population fluctuations correlated to other factors such as host fruiting phenology^26,30^.

Mexican fruit fly has been considered a potential threat to US agriculture for decades. In the 1930s, Darby and Kapp^31^, and later Flitters and Messenger^32^ used programmable bioclimatic cabinets to estimate the fly’s invasive potential in temperate regions. Flitters and Messenger^32^ tested survival using weather profiles from several locations in the USA, and found that the fly is limited by high temperatures and less by low humidity. The fly occupies relatively frost free areas, has a long lifespan, lays 2000-3000 eggs, and begins oviposition above 12.78°C after a sexual maturation period of 10 and 15 days, (see ^22,32^). Tejeda et al.^33^ found that *A*. *ludens* adapts rapidly to dry environments through increased desiccation resistance, an extended lifespan, and longer pupal development time and delayed reproduction. It has a larger mass (11–20 mg) compared to other fruit flies, a 3fold lipid accumulation, and has a decreased surface-to-volume ratio that are thought part of its evolutionary response that increases desiccation resistance^33^.


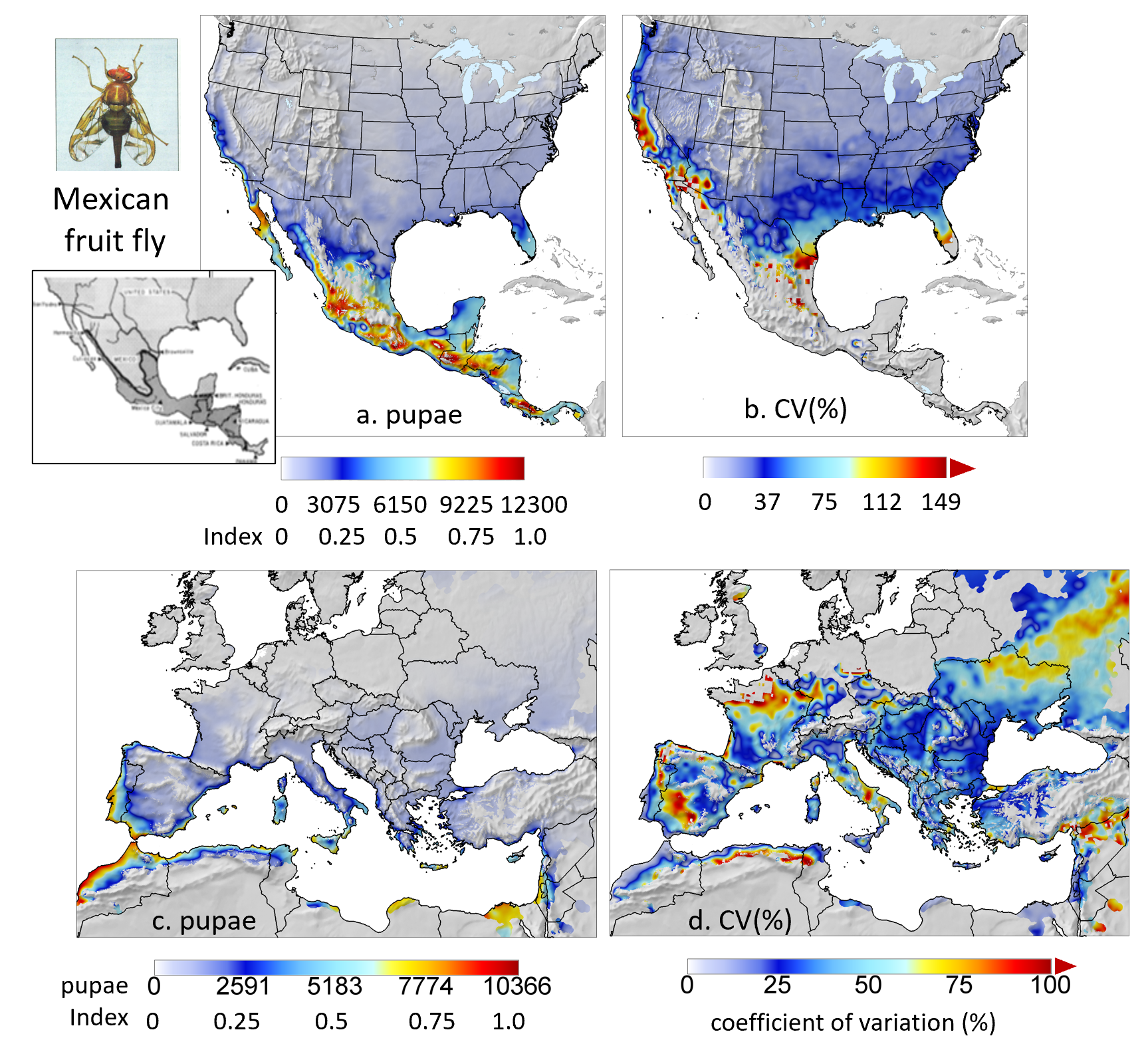


Figure S6. Average prospective distribution of **Mexican fruit fly** in North and Central America (NA-CA) and the European-Mediterranean region (E-M) for the period 1980-1990: (NA-CA):

(a) sum of annual pupae, (b) CV(%), (E-M) (c) sum of annual pupae, and (d) CV(%). The inset in (a) is from Messenger and Flitters^34^. Clip of Mexican fruit fly was taken from a photograph by Jack Kelly Clark provided courtesy of the University of California Statewide IPM Program.

**Prospective distribution of Mexican fruit fly -** The prospective area of favorability in North America predicted by the model is similar to the area roughly mapped by Flitters and Messenger^32^ (Fig. S6a inset). The V-shaped gap in the Messenger Flitters inset map is the mountainous areas of Central Mexico. The areas of highest climatic favorability are the frost-free areas of Central Mexico through Central America and parts of Baja California (Index>0.6). The lower elevation of Mexico and the coastal region of Southern California have low favorability (normalized pupal density indices ($\text{FI}\in[0.25,0.5]$). In contrast to most of Mexico, areas with high coefficients of variability for pupal densities occur throughout California except in small areas of near coastal southern California (Fig. 6b). The Great Central Valley of California is unfavorable with FI<0.25 due very high summer temperatures, and recurring low winter temperatures with winter frosts in many areas that combined result in high CV(%) for predicted mexfly pupal densities (Fig. S6b).

Based on bioclimatic studies, Messenger and Flitters^34^ and Flitters and Messenger^32^ predicted the fly could develop year around at Compton (Los Angeles) and Chula Vista (San Diego) in the southern Californian coastal plain (FI ~ 0. 5), and in climates like Brownsville in south Texas (FI <0.3), and Orlando in south central Florida (FI ~ 0.5). Only their prediction for Brownsville, TX in the lower Rio Grande Valley is sharply at odds with our prospective distribution of the fly, and yet it is the only location that has frequent detectable mexfly infestations despite the high CV for predicted pupal densities in south Texas. At Zapata and Brownsville TX, the fly is known to overwinter as immature stages in sour orange in urban garden microclimates as suggested by the trapping of immature adults in early spring (D. Thomas pers. comm.). The continued presence of mexfly in the southern tip of Texas could be due its relative proximity to endemic areas of Mexico, introduction of infested fruit in cars and produce trucks, and the fly’s dispersal ability^35^ on the weather system locally called the Mexican Trumpet - a steady air flow current from the Sierra Madre that sweeps northward into Texas between Del Rio and Eagle Pass (D. Thomas, pers. comm.). Further, as found for the North American screwworm, dispersal could be aided by strong southerly summer North American monsoon winds from Mexico that carry aeroplankton northward to Arizona, New Mexico, and especially to south Texas^36^. The vast desert areas of Arizona, southern California and NW Mexico with inhospitable high temperatures and low RH would appear to be a natural barrier to the fly.

The effects of temperature are summarized by a multiple regression model of total average pupae >10 per year on the average of $\bar{\mu}_{<10.3C}$ (t-value = -6.45), $\bar{\mu}_{>32^{\circ}C}$ (t = -23.12), and their *CVs* (left superscript)

${}^{CV}\bar{\mu}_{<10.3^{\circ}C}$ (t = 5.40) and ${}^{CV}\bar{\mu}_{>32^{\circ}C}$ (t < 2) across all years [eqn. S3]. Only ${}^{CV}\bar{\mu}_{>32^{\circ}C}$is not significant.

$\begin{matrix} total\text{ }pupae\text{ (>10)}=\text{5949.4 }-317.9\bar{\mu}_{<10.3^{\circ}C}-1629.0\bar{\mu}_{>32^{\circ}C}+35.12\text{ }{}^{CV}\bar{\mu}_{<10.3^{\circ}C} \\ \text{ }R^{2}=0.83,\text{ }df=\text{ }177,\text{ }F=283.08 \end{matrix}$ [S3]

The area of greatest favorability is circumscribed by the isoline $\bar{\mu}_{<10.3C}$~1.2 and variability ${}^{CV}\bar{\mu}_{<10.3C}$< 80%. This conforms to early assessments of fly’s low tolerance to cold^31,32^. Hot weather is also a limiting factor ($\bar{\mu}_{>32^{\circ}C}$), particularly in the Central Valley and desert areas of southern California, and Arizona, and northeastern Mexico with CV >80% (Fig. S6b).

The Mexican fruit fly has not been reported in the European-Mediterranean region, where the prospective distribution of favorability is restricted to frost free areas, with greatest favorability predicted in SW Portugal, Spain, coastal Morocco, Egypt, and Israel, and less along the North African coast, eastern coastal Spain, Sardinia, Sicily, and other warmer areas (Fig. S6c, d).

**Climate change effects on geographic distribution and relative abundance**

From the perspective of a poikilotherm organism, climate change weather is another weather pattern that may or may not be favorable for the species. PBDMs are mechanistic descriptions of the biology that is assumed unchanged except on an evolutionary time scale^37^, and hence can be used to assess the effects of climate change on the distribution and relative abundance of ectotherm species in time and geographic space.

**Medfly -** The PBDM for medfly suggests that with climate change, favorability will decrease in Central America and Mexico due to increased temperatures, while favorability will increase in Baja and US California, as coastal California becomes more tropical (Fig. S1 vs. S7). In the Palearctic region, the potential area of favorability for medfly moves northward in some areas with increased favorability in the Levant, but declines in other areas such as the Nile Delta due to high temperature (Fig. S1 vs. S7).


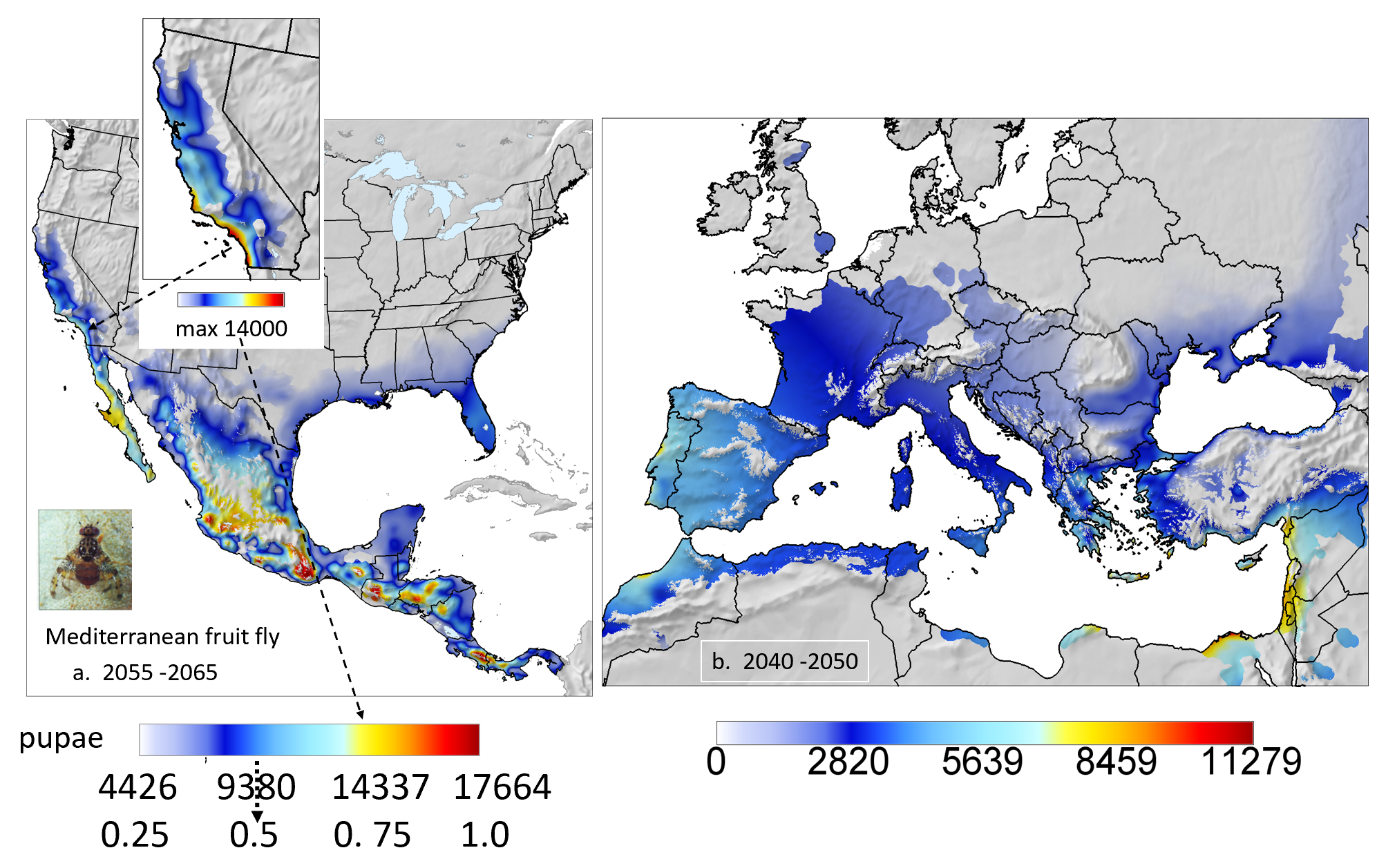


Figure S7. Climate change average prospective distribution of Mediterranean fruit fly in North and Central America (2055-65) with insets for California, and in the European-Mediterranean region (2040-50). Clip of Mediterranean fruit fly was taken from a photograph by Jack Kelly Clark provided courtesy of the University of California Statewide IPM Program.

**Melon fly -** The distribution of melon fly will prospectively increase in Central America and along the Gulf Coast of the USA, with conditions predicted to become more favorable in NW Mexico, part of southern Baja California and coastal California. In the European-Mediterranean region, the area of favorability will increase (Fig. S2 vs S8) with the Nile Delta and the Levant becoming more hospitable. Note that the maximum densities in Mexico and Central America are nearly double those in the most favorable areas of the Mediterranean Basin (e.g., the Nile Delta)


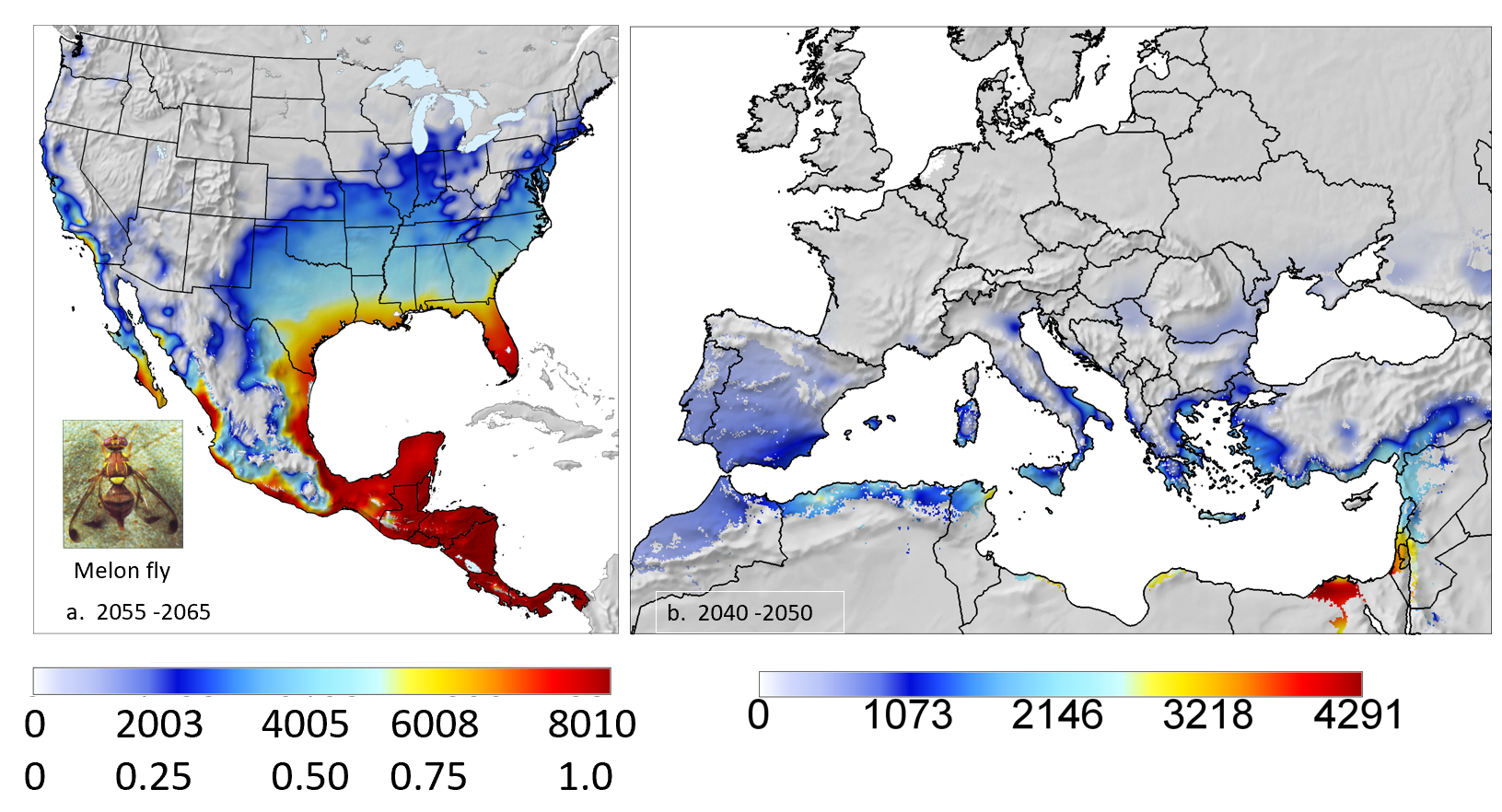


Figure S8. Climate change average prospective distribution of melon fruit fly in North and Central America (2055-65), and in the European-Mediterranean region (2040-50). Clip of melon fly was taken from a photograph by Jack Kelly Clark provided courtesy of the University of California Statewide IPM Program.

**Oriental Fruit fly -** The range of the oriental fruit fly is projected to increase in Mexico and Central America, and in southern Florida, and increase slightly in NW Mexico (Baja California), and southern California (Fig. S5 vs S9). In the Mediterranean Basin, areas of highest prospective favorability are principally in Egypt and the Levant with increased favorability in Morocco (Fig. 5 vs S9). Note that highest densities in Mexico-Central America are ~70% greater than in the Nile delta of Egypt.


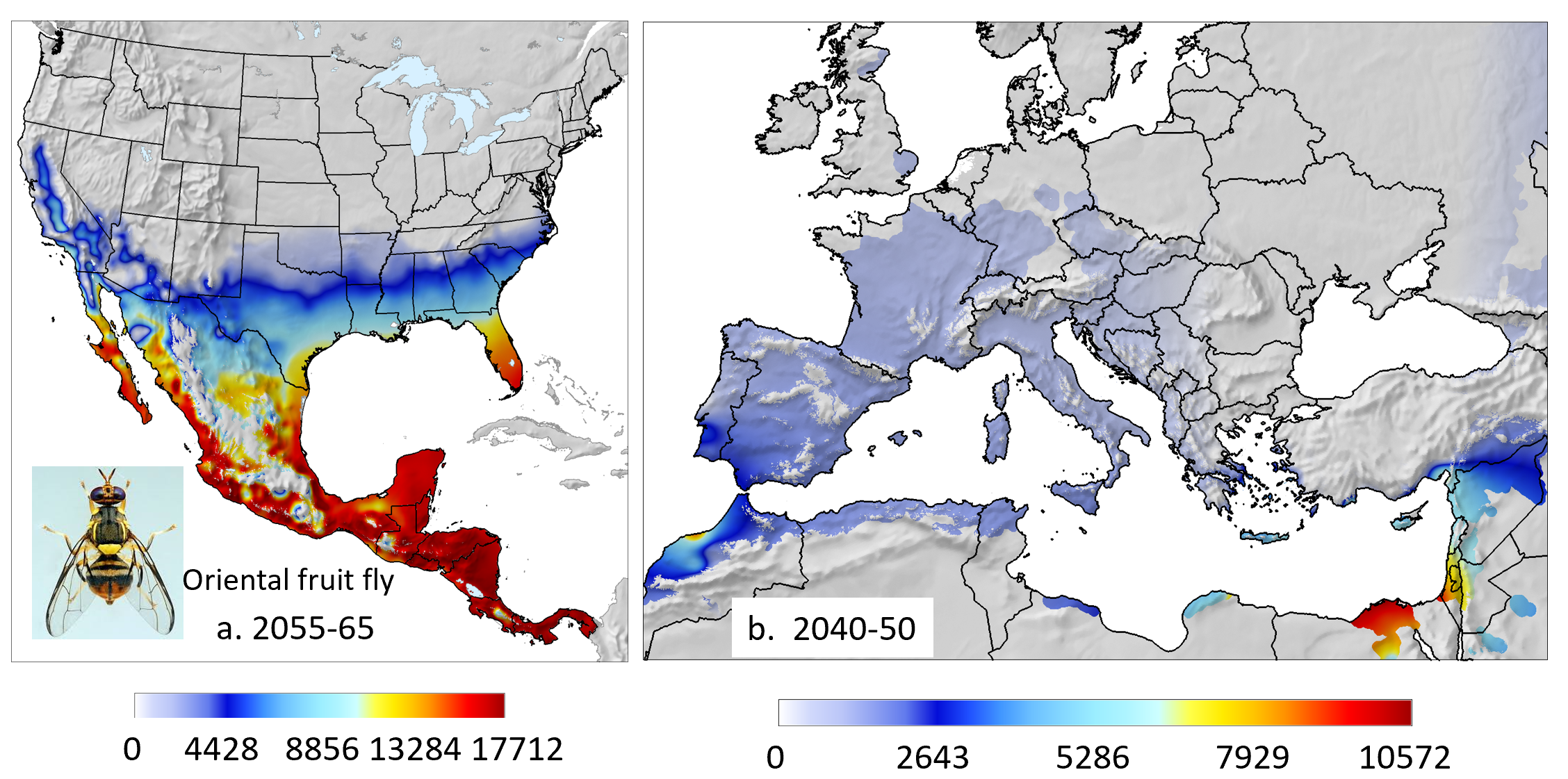


Figure S9. Climate change: average prospective distribution of oriental fruit fly in (a) North and Central America (2055-65), and (b) in the European-Mediterranean region (2040-50). Clip of oriental fruit fly was taken from a photograph by Jack Kelly Clark provided courtesy of the University of California Statewide IPM Program.

**Mexican fruit fly –** The area of favorability for the Mexican fruit fly is projected to contract in Mexico and Central America with increased range expansion in Baja California and near coastal California (Figure S6 vs. S10).

Its prospective range will expand in western Portugal, central Spain, and Crete, but contract in Morocco, along the north African coast and the Levant (Fig. 10b).


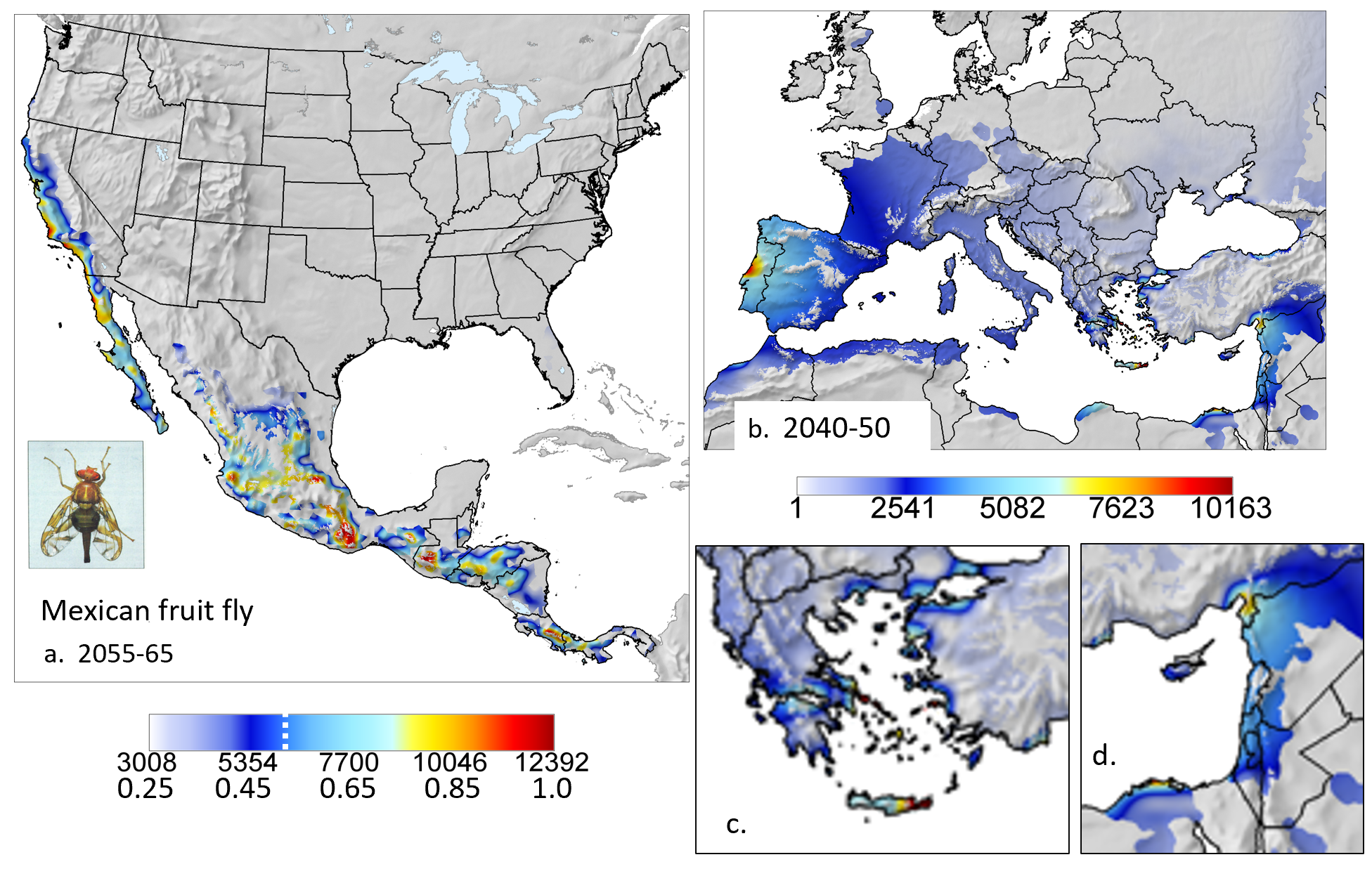


Figure S10. Climate change average prospective distribution of Mexican fruit fly in (a) North and Central America (2055-65), and (b) in the European-Mediterranean region (2040-50) with insets for Greece and the Levant. Clip of Mexican fruit fly was taken from a photograph by Jack Kelly Clark provided courtesy of the University of California Statewide IPM Program.

**Discussion of the distributed maturation time model (Manetsch**^38^ **(1976) and Vansickle**^39^ **(1977))**

Cohorts of individuals in a population have different developmental rate due to intrinsic differences, and may experience different weather, and hence in the aggregate will have different patterns of time-varying mean developmental times and variance. Without specifying the cause, this can easily modeled using the highly accessible weather-driven distributed maturation time model of Manetsch^38^ and Vansickle^39^ (eqn. S5, Fig. S11). We note that other dynamics models could also be used ^40,41^.


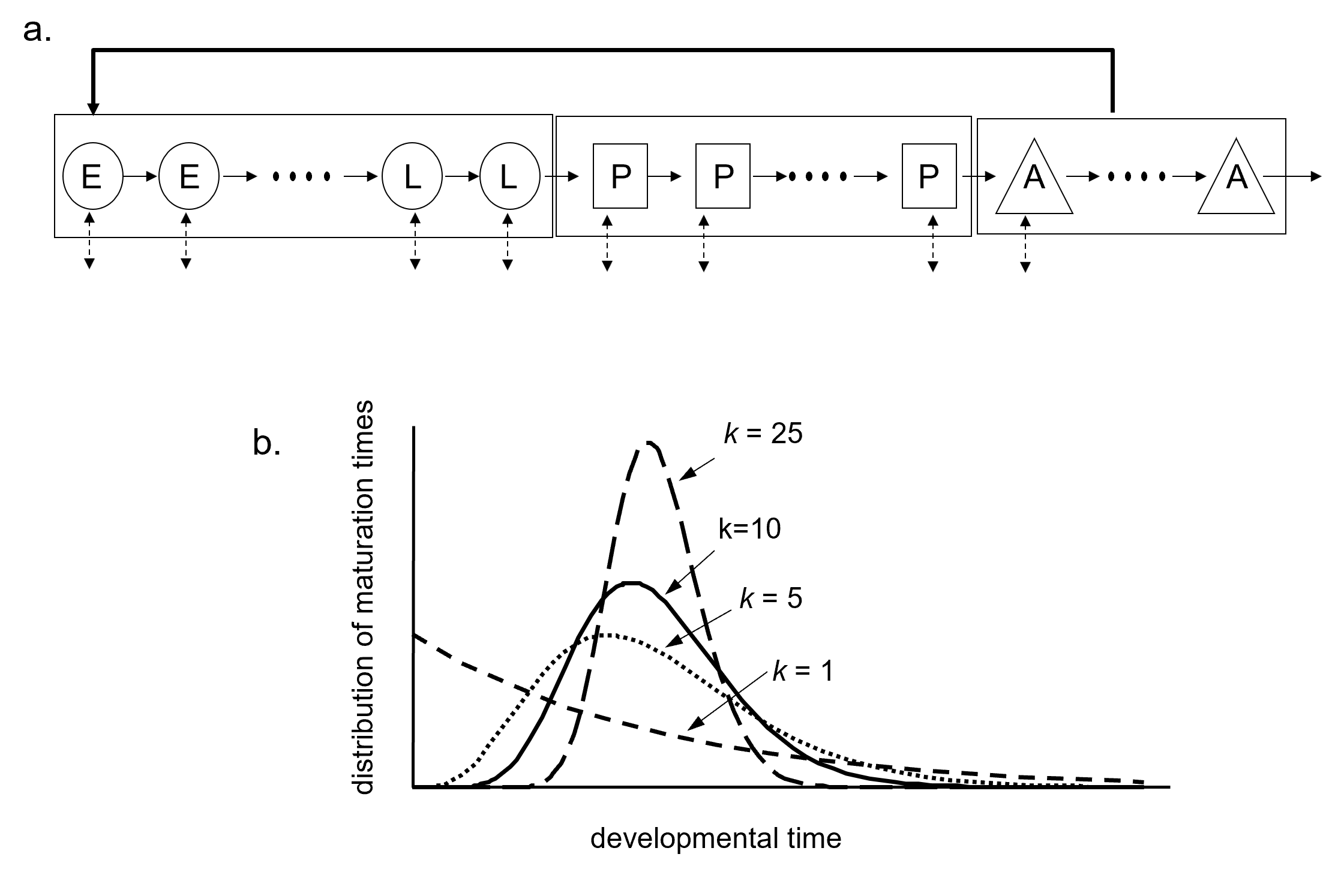


**Figure S11.** An age structured population dynamics model for the dynamics: (11a) for the egg (symbol *E*), larval (*L*), pupal (*P*) and adult (*A*) stages with flows (aging) between age classes and stages, the double arrows indicate net age-specific mortality, and (11b) the stylized distribution of developmental times based on the number of age cohorts (k) in each stage in figure 2a (see refs.^42,43^).

There are four life stages (left superscript s = E, L, P, A for the egg, larval, pupal, and adult stages respectively, Figure S11a), and are modeled by a system of *^s^k* equations (e.g., eqn. [S5]) that describes the dynamics of the *i^th^* of *^s^k* age class. The distribution of developmental times in the absence of mortality is described by an Erlang distribution with parameter *k* equal to the number of within stage age classes required to reproduce the observed mean (Δ) and variance (var) of developmental time of cohort members (Figure S11b). Using the egg stage (left super script E) as our foil, the dynamics are modeled as rates (*^E^r_i_,* i=1, *^E^k*)

[S5]

The instantaneous solutions of eqns. S5 are eqns. S6.

[S6]

The other life stages (and species) may be similarly modeled with the outflow from the k^th^ age class of the previous stage flowing into the first age class of the next stage (e.g., egg to larval, etc.), with final death of surviving adults being outflows from the last age class *^A^r_k_*.

Ignoring the stage superscript, *r_i_* is the number density of the *i*th age class, *dt* is the change in time (e.g., a day), Δ is the expected mean stage developmental time, and *μ*_i_(*t*) is the proportional age-specific net loss rate as modified by temperature, age, net migration, and mortality due to natural enemies. N.B. The units in our model are in number units, but mass and other units could be easily accommodated. In the discretized form, Δ*_x_(T(t))* is the daily increment of physiological age in degree days for the life stage. The Euler’s integration subroutine written in Pascal for the discretized model is found in Gutierrez^42^ (page 157).

**Table S1.** Summary of biodemographic functions and parameters for the tephritid olive fly and the spotted wing drosophila.

| BDF/parameters | olive fly | spotted wing drosophila |
| --- | --- | --- |
| *developmental-times*  egg-larvae | 243.55dd > 8°C | 141.0dd > 5.975°C |
| pupae | 248dd > 8°C | 92.2dd >5.975°C |
| adults | 1020dd > 8°C | 1200dd >5.975°C |
| quiesent adults | 1500dd > 8°C | 1275dd >5.975°C |
| ^E-L^R(T)  ^P-A^Δ_x_(T) | 0.0041(*T* - 8)/(1+ 6.5*^T^*^-34^)  ^P-A^Δ_x_(*T*) = ^E-L^Δ_x_(*T*) | 0.0042 (*T*-5.975)/(1+4.5 *^T^*^-31.5^) for E-P  ^A^Δ_x_(*T*) = ^E-P^Δ_x_(*T*) |
| ^E-L^k, ^P^k, ^A^k | 40, 40, 60 | 25, 25, 25 |
| *f(x)* | $\begin{matrix} \text{if }x\geq\text{8 then } \\ \text{9.5(}x\text{-8)/(1.075}^{\text{(}x\text{-8)}})\text{ } \end{matrix}$ | if *x* > 1 then  6.0*x*/(1.0475 *^x^*) at *T* = 21°C |
| 0 < φ_T_ <1 | *F*(*T*, 12, 33) | *F*(*T*, 12.75, 31.0) |
| 0 < φ_RH_ <1 | 1 -0.000000123*RH*^4^ + 0.00001579*RH*^3^ - 0.00048760*RH*^2^ + 0.01168474*RH* + 0.093554 | 1.0 - 42.895*RH* ^-1.759^ |
| 0< ^E-P^*μ* <1  0< ^A^*μ*<1 | $\left\{\begin{matrix} \text{if }T\leq\text{ 0 then 0.0125e}^{\text{-0.438}T} \\ \text{if 0<}T\leq\text{ 29 then }0.01 \\ \text{if }T\text{ >29 then 0.0125e}^{\text{0.25*(}T\text{-30)}} \end{matrix}\right.$ ^A^*μ* = ^E-P^*μ* | 0.0000022983*T*^4^ +0.0001609536*T*^3^  + 0.0041913761*T*^2^ - 0.0466960983*T* + 0.2006769449  ^A^*μ* = ^E-P^*μ* |

**T** = mean temperature, **RH** = mean % relative humidity, **k** = stage specific Erlang parameter in text eqn. 1. Scripts **E, L, P, A** = egg, larval, pupal, and adult stages, ^stage^R(T) = developmental rate, ***f(x)* =** *per capita* reproductive rate per day on age (*x*) at 25°C, **^stage^Δ** = developmental time in dd above the lower threshold, **^stage^Δ_x_** = (stage developmental time multiplied by stage developmental rate), **0 < *F*(*T*** or ***RH*, min, max) < 1** is a symmetrical function between min and max and equal 1 at (max + min)/2)**. φ_RH_** and **φ_T_** are scalars for reproduction, and ***μ*_stage_** is the temperature-dependent stage mortality rate. ***φ_RH_*** for olive fly had not previously been published.^43^

**Supplementary references**

1. De Meyer, M., Robertson, M. P., Peterson, A. T. & Mansell, M. W. Ecological niches and potential geographical distributions of Mediterranean fruit fly (*Ceratitis capitata*) and Natal fruit fly (*Ceratitis rosa*). *J. Biogeogr.* **35**, 270–281 (2008).

2. Vail, P. V., Moore, I. & Nadel, D. The Mediterranean fruit fly in Central America. *IAEA Bull.* **18**, 42–46 (1976).

3. Enkerlin, W. R. *et al.* The Moscamed regional programme: review of a success story of area-wide sterile insect technique application. *Entomol. Exp. Appl.* **164**, 188–203 (2017).

4. Gutierrez, A. P. & Ponti, L. Assessing the invasive potential of the Mediterranean fruit fly in California and Italy. *Biol. Invasions* **13**, 2661–2676 (2011).

5. Papadopoulos, N. T., Plant, R. E. & Carey, J. R. From trickle to flood: the large-scale, cryptic invasion of California by tropical fruit flies. *Proc. R. Soc. Biol. Sci. Ser. B* **280**, 20131466 (2013).

6. Suckling, D. M. *et al.* Eradication of tephritid fruit fly pest populations: outcomes and prospects. *Pest Manag. Sci.* **72**, 456–65 (2016).

7. McInnis, D. O. *et al.* Can polyphagous invasive tephritid pest populations escape detection for years under favorable climatic and host conditions? *Am. Entomol.* **63**, 89–99 (2017).

8. Carey, J. R., Papadopoulos, N. & Plant, R. The 30‐year debate on a multi‐billion‐dollar threat: tephritid fruit fly establishment in California. *Am. Entomol.* **63**, 100–113 (2017).

9. Szyniszewska, A. M. *et al.* CLIMEX and MED-FOES models for predicting the variability in growth potential and persistence of Mediterranean fruit fly (Diptera: Tephritidae) populations. *Ann. Entomol. Soc. Am.* **113**, 114–124 (2020).

10. Vera, M. T., Rodriguez, R., Segura, D. F., Cladera, J. L. & Sutherst, R. W. Potential geographical distribution of the Mediterranean fruit fly, *Ceratitis capitata* (Diptera: Tephritidae), with emphasis on Argentina and Australia. *Environ. Entomol.* **31**, 1009–1022 (2002).

11. Israely, N., Ritte, U. & Oman, S. D. Inability of *Ceratitis capitata* (Diptera: Tephritidae) to Overwinter in the Judean Hills. *J. Econ. Entomol.* **97**, 33–42 (2004).

12. Israely, N., Yuval, B., Kitron, U. & Nestel, D. Population fluctuations of adult Mediterranean fruit flies (Diptera: Tephritidae) in a Mediterranean heterogeneous agricultural region. *Environ. Entomol.* **26**, 1263–1269 (1997).

13. Papadopoulos, N. T., Carey, J. R., Katsoyannos, B. I. & Kouloussis, N. A. Overwintering of *Ceratitis capitata* (Diptera: Tephritidae) in northern Greece. *Ann. Entomol. Soc. Am.* **89**, 526–534 (1996).

14. Rigamonti, I. E. Contributions to the knowledge of *Ceratitis capitata* Wied. (Diptera, Tephritidae) in Northern Italy II. Overwintering in Lombardy. *Boll. Zool. Agrar. E Bachic.* **Ser. II, 36**, 101–116 (2004).

15. Fitzpatrick, E. A. & Nix, H. A. The climatic factor in Australian grasslands ecology. in *Australian Grasslands* (ed. Moore, R. M.) 3–26 (Australian National University Press, 1970).

16. Gutierrez, A. P., Nix, H. A., Havenstein, D. E. & Moore, P. A. The ecology of *Aphis craccivora* Koch and subterranean clover stunt virus in south-east Australia. III. A regional perspective of the phenology and migration of the cowpea aphid. *J. Appl. Ecol.* **11**, 21–35 (1974).

17. CABI. *Bactrocera dorsalis*. *Crop Prot. Compend. CAB Int. Wallingford UK* (2020).

18. Goergen, G., Vayssieres, J. F., Gnanvossou, D. & Tindo, M. *Bactrocera invadens* (Diptera: Tephritidae), a new invasive fruit fly pest for the Afrotropical Region: host plant range and distribution in West and Central Africa. *Environ. Entomol.* **40**, 844–54 (2011).

19. Vargas, R. I. *et al.* Area-wide suppression of the Mediterranean fruit fly, *Ceratitis capitata*, and the Oriental fruit fly, *Bactrocera dorsalis*, in Kamuela, Hawaii. *J. Insect Sci.* **10**, 135 (2010).

20. Zhao, Z. *et al.* The failure of success: cyclic recurrences of a globally invasive pest. *Ecol. Appl.* **29**, e01991 (2019).

21. Stephens, A. E., Kriticos, D. J. & Leriche, A. The current and future potential geographical distribution of the oriental fruit fly, *Bactrocera dorsalis* (Diptera: Tephritidae). *Bull. Entomol. Res.* **97**, 369–78 (2007).

22. Aluja, M. Bionomics and management of *Anastrepha*. *Annu. Rev. Entomol.* **39**, 155–178 (1994).

23. Ruiz-Arce, R., Owen, C. L., Thomas, D. B., Barr, N. B. & McPheron, B. A. Phylogeographic structure in *Anastrepha ludens* (Diptera: Tephritidae) populations inferred with mtDNA sequencing. *J. Econ. Entomol.* **108**, 1324–36 (2015).

24. Norrbom, A. L. & Foote, R. H. The taxonomy and zoogeography of the genus *Anastrepha* (Diptera: Tephritidae). in *Fruit Flies. Their Biology, Natural Enemies and Control* (eds. Robinson, A. S. & Hooper, G.) 15–26 (Elsevier, 1989).

25. Thomas, D. B. Reproductive phenology of the Mexican fruitfly, *Anastrepha ludens* (Loew) (Diptera Tephritidae) in the Sierra Madre Oriental, Northern Mexico. *Neotrop. Entomol.* **32**, 385–397 (2003).

26. Thomas, D. B. Mexican fruit fly (Diptera: Tephritidae) and the phenology of its native host plant yellow chapote (Rutaceae) in Mexico. *J. Entomol. Sci.* **47**, 1–16 (2012).

27. Celedonio-Hurtado, H., Aluja, M. & Liedo, P. Adult population fluctuations of *Anastrepha* sp. (Diptera: Tephritidae) in tropical habitats of Chiapas, Mexico. *Environ. Entomol.* **24**, 861–869 (1995).

28. Aluja, M. *et al.* Seasonal population fluctuations and ecological implications for the management of *Anastrepha* fruit flies (Diptera: Tephritidae) in commercial mango orchards in southern Mexico. *J. Econ. Entomol.* **89**, 654–667 (1996).

29. Sequeira, R., Millar, L. & Bartels, D. *Identification of susceptible areas for the establishment of* Anastrepha *spp. fruit flies in the United States and analysis of selected pathways*. (USDA Internal document, The Center for Plant Health Science and Technology, 2001).

30. Montoya, P., Flores, S. & Toledo, J. Effect of rainfall and soil moisture on survival of adults and immature stages of *Anastrepha ludens* and *A. obliqua* (Diptera: Tephritidae) under semi-field conditions. *Fla. Entomol.* **91**, 643–650 (2008).

31. Darby, H. H. & Kapp, E. M. Observations on the thermal death points of *Anatrepha ludens* (Loew). *U. S. Dep. Agric. Tech. Bull.* **400**, (1933).

32. Flitters, N. E. & Messenger, P. S. Effect of temperature and humidity on development and potential distribution of the Mexican fruit fly in the United States. *U. S. Dep. Agric. Tech. Bull.* **1330**, (1965).

33. Tejeda, M. T. *et al.* Reasons for success: Rapid evolution for desiccation resistance and life-history changes in the polyphagous fly *Anastrepha ludens*. *Evolution* **70**, 2583–2594 (2016).

34. Messenger, P. S. & Flitters, N. E. Bioclimatic studies of the Mexican fruit fly. *Calif. Avocado Soc. Yearb.* **41**, 119–127 (1957).

35. Fletcher, B. S. Ecology: movements of tephritid fruit flies. in *World Crop Pests, Vol. 3B: Fruit Flies: Their Biology, Natural Enemies and Control.* (eds. Robinson, A. S. & Hooper, G.) 209–219 (Elsevier, 1989).

36. Gutierrez, A. P., Ponti, L. & Arias, P. A. Deconstructing the eradication of new world screwworm in North America: retrospective analysis and climate warming effects. *Med. Vet. Entomol.* **33**, 282–295 (2019).

37. Bennett, J. M. *et al.* The evolution of critical thermal limits of life on Earth. *Nat. Commun.* **12**, 1198 (2021).

38. Manetsch, T. J. Time-varying distributed delays and their use in aggregative models of large systems. *IEEE Trans. Syst. Man Cybern.* **6**, 547–553 (1976).

39. Vansickle, J. Attrition in distributed delay models. *IEEE Trans. Syst. Man Cybern.* **7**, 635–638 (1977).

40. Buffoni, G. & Pasquali, S. Structured population dynamics: continuous size and discontinuous stage structures. *J. Math. Biol.* **54**, 555–595 (2007).

41. Di Cola, G., Gilioli, G. & Baumgärtner, J. Mathematical models for age-structured population dynamics. in *Ecological entomology* (eds. Huffaker, C. B. & Gutierrez, A. P.) (Wiley, 1999).

42. Gutierrez, A. P. *Applied population ecology: a supply-demand approach*. (John Wiley and Sons, 1996).

43. Severini, M., Alilla, R., Pesolillo, S. & Baumgärtner, J. Fenologia della vite e della *Lobesia botrana* (Lep. Tortricidae) nella zona dei Castelli Romani. *Riv. Ital. Agrometeorol.* **3**, 34–39 (2005).
