# Supplementary material for: Invasive potential of tropical fruit flies in temperate regions under climate change
Source: Commun Biol. 2021 Sep 30;4:1141. doi: 10.1038/s42003-021-02599-9 (PMC8484444; doi:10.1038/s42003-021-02599-9)
Supplement: Supplementary file 3 — Description of Additional Supplementary Files [file 42003_2021_2599_MOESM3_ESM.pdf]

Description of Additional Supplementary Files

Filename: Supplementary Data 1
Description: Source data for the oriental fruit fly

Filename: Supplementary Data 2
Description: Source data for the Mexican fruit fly

Filename: Supplementary Data 3
Description: Source data for the melon fruit fly

Filename: Supplementary Data 4
Description: Source data for the Mediterranean fruit fly
