# Supplementary material for: Invasive potential of tropical fruit flies in temperate regions under climate change
Source: Commun Biol. 2021 Sep 30;4:1141. doi: 10.1038/s42003-021-02599-9 (PMC8484444; doi:10.1038/s42003-021-02599-9)
Supplement: Supplementary file 8 — Reporting Summary [file 42003_2021_2599_MOESM8_ESM.pdf]

nature research

Corresponding author(s): COMMSBIO-21-0038A

Last updated by author(s): 2021/03/29

Reporting Summary

Nature Research wishes to improve the reproducibility of the work that we publish. This form provides structure for consistency and transparency in reporting. For further information on Nature Research policies, see our Editorial Policies and the Editorial Policy Checklist.

Statistics

For all statistical analyses, confirm that the following items are present in the figure legend, table legend, main text, or Methods section.

n/a	Confirmed	
☒	☐	The exact sample size (n) for each experimental group/condition, given as a discrete number and unit of measurement
☒	☐	A statement on whether measurements were taken from distinct samples or whether the same sample was measured repeatedly
☒	☐	The statistical test(s) used AND whether they are one- or two-sided<br>*Only common tests should be described solely by name; describe more complex techniques in the Methods section.*
☒	☐	A description of all covariates tested
☒	☐	A description of any assumptions or corrections, such as tests of normality and adjustment for multiple comparisons
☐	☒	A full description of the statistical parameters including central tendency (e.g. means) or other basic estimates (e.g. regression coefficient) AND variation (e.g. standard deviation) or associated estimates of uncertainty (e.g. confidence intervals)
☐	☒	For null hypothesis testing, the test statistic (e.g. F, t, r) with confidence intervals, effect sizes, degrees of freedom and P value noted<br>*Give P values as exact values whenever suitable.*
☒	☐	For Bayesian analysis, information on the choice of priors and Markov chain Monte Carlo settings
☒	☐	For hierarchical and complex designs, identification of the appropriate level for tests and full reporting of outcomes
☒	☐	Estimates of effect sizes (e.g. Cohen's d, Pearson's r), indicating how they were calculated

Our web collection on statistics for biologists contains articles on many of the points above.

Software and code

Policy information about availability of computer code

Data collection	N/A -- all of the data were estimated from the literature.
Data analysis	The data used to parameterize the biodemographic functions (BDF, Tables 2 and 3) of the weather-driven population dynamics fruit fly models were gleaned from the wide variety of sources cited in the text (see methods). Polynomial fits to data for some BDFs were made using the Microsoft EXCEL add trendline functions, while non-linear developmental rate and oviposition profile functions were fit using EXCEL Analysis ToolPAK and iteratively. The 3-dimentional figures of adult fly fecundity on age and temperature were fit to BDFs functions using MATLAB R2019. The numerical models were coded in Borland Delphi Pascal 3 Professional, and are embedded in a large system base developed over three decades for handling large weather files and input-output data used to run the models. The core algorithm for the Euler integration of the distributed maturation time population dynamics model (text equation 1) with and without age specific attrition used in our analysis has been published (reference 12, Gutierrez 1996, page 157). The sources of the weather data were outlined in the methods section. The system comprises PBDMs for 30+ species of plants, herbivores, parasitoids, predators, and pathogens published as PBDM analyses based on the same methods (e.g., text references). The system is managed by the nonprofit scientific consortium Center for the Analysis of Sustainable Agricultural Systems Global (http://www.casasglobal.org/). The fruit fly system is currently being recoded in Python using an object-oriented programming paradigm for release as open source. Multiple regression summaries of the simulation results reported in the Supplemental Information were made using the open-source R language and environment for statistical computing (R Foundation for Statistical Computing, Vienna, Austria; URL http://www.R-project.org). All GIS maps were developed using the open-source Geographic Resources Analysis Support System (GRASS 7.9, https://grass.osgeo.org).

For manuscripts utilizing custom algorithms or software that are central to the research but not yet described in published literature, software must be made available to editors and reviewers. We strongly encourage code deposition in a community repository (e.g. GitHub). See the Nature Research guidelines for submitting code & software for further information.

Data

Policy information about availability of data

All manuscripts must include a data availability statement. This statement should provide the following information, where applicable:

- Accession codes, unique identifiers, or web links for publicly available datasets
- A list of figures that have associated raw data
- A description of any restrictions on data availability

All of the data used in the analyses were presented in method figures and as fitted functions in tables 2 and 3.

Field-specific reporting

Please select the one below that is the best fit for your research. If you are not sure, read the appropriate sections before making your selection.

☐ Life sciences ☐ Behavioural & social sciences ☒ Ecological, evolutionary & environmental sciences

For a reference copy of the document with all sections, see nature.com/documents/nr-reporting-summary-flat.pdf

Ecological, evolutionary & environmental sciences study design

All studies must disclose on these points even when the disclosure is negative.

Study description	The study brings together data from disparate studies reported in the literature to estimate the effects of extant and climate change weather of the geographic distribution and abundance of four invasive tropical fruit flies. No studies with live organisms were conducted. The study developed biodemographic relationships that characterize the response of the four species to weather.
Research sample	N/A
Sampling strategy	N/A
Data collection	N/A
Timing and spatial scale	N/A
Data exclusions	Data on the same biological attributes such as the effects of temperature on the rate of development were found in the literature , but the data were often incomplete, and hence data from the multiple sources were pooled to estimated the function. Only data from Kandakoor et al. (ref 89) were excluded because they varied markedly from those reported by other authors.
Reproducibility	N/A
Randomization	N/A
Blinding	N/A

Did the study involve field work? ☐ Yes ☒ No

Reporting for specific materials, systems and methods

We require information from authors about some types of materials, experimental systems and methods used in many studies. Here, indicate whether each material, system or method listed is relevant to your study. If you are not sure if a list item applies to your research, read the appropriate section before selecting a response.

Materials & experimental systems

n/a	Involved in the study	
☒	☐	Antibodies
☒	☐	Eukaryotic cell lines
☒	☐	Palaeontology and archaeology
☒	☐	Animals and other organisms
☒	☐	Human research participants
☒	☐	Clinical data
☒	☐	Dual use research of concern

Methods

n/a	Involved in the study	
☒	☐	ChIP-seq
☒	☐	Flow cytometry
☒	☐	MRI-based neuroimaging
